# Geospatial evaluation of San Francisco, California’s homeless encampment sweeps injunction

**DOI:** 10.3389/fpubh.2025.1516105

**Published:** 2025-02-05

**Authors:** Sean G. Young, Vincent Truong, William P. Watson, Cari A. Bogulski

**Affiliations:** ^1^Peter O’Donnell Jr. School of Public Health, University of Texas Southwestern Medical Center, Dallas, TX, United States; ^2^Department of Health Policy and Management, Fay W. Boozman College of Public Health, University of Arkansas for Medical Sciences, Little Rock, AR, United States; ^3^Department of Biomedical Informatics, College of Medicine, University of Arkansas for Medical Sciences, Little Rock, AR, United States

**Keywords:** homelessness, encampments, public policy, 311 data, cluster detection, hot spot analysis

## Abstract

**Introduction:**

Homelessness remains a pervasive issue in many communities within the United States (US). “Sit-lie” policies restrict where individuals can sit or lie down in public places and are frequently passed and cited to forcibly re-locate individuals experiencing homelessness. In December 2022, a federal judge issued a temporary injunction of San Francisco, California’s sit-lie policy, due to a pending lawsuit arguing that the enforcement of such policies when shelter cannot be offered is a violation of the Eighth Amendment of the US Constitution.

**Methods:**

To examine the effects of this preliminary injunction, we spatially examined data from San Francisco’s 311 reporting system to identify encampment report hot spots.

**Results and discussion:**

Overall, we found spatial shifting of encampment reporting, but fewer reports overall during the preliminary injunction period, relative to 1 year prior. Future work should examine the effect of the reversal of the injunction following a recent Supreme Court decision and subsequent ruling by the Ninth Circuit Court of Appeals.

## 1 Introduction

State and local policies addressing homelessness in public spaces are often controversial and have an impact on public health. Some such policies include anti-camping ordinances, anti-sleeping ordinances, and ordinances prohibiting sitting or lying down in public places (henceforth “sit-lie” policies). Proponents of such policies argue that they encourage movement to safer, indoor spaces (1). Urban encampments pose documented risks to public health, lacking many of the resources to meet basic human needs such as sanitation, safety, and access to clean water (2, 3).

Opponents argue that these policies directly harm people experiencing homelessness (4). Evidence suggests that there are negative consequences to the enforcement of such policies on people experiencing homelessness, such as a loss of resources when belongings are destroyed or relocated; increased fear, anxiety, stress, anger, and worry; and increased re-location to more (rather than less) dangerous places (1, 5–7). There is also evidence of increased risk of crime victimization for people experiencing homelessness (8, 9). However, many parts of the United States (US) lack sufficient housing for everyone experiencing homelessness, either in the form of emergency shelters, transitional housing beds, or permanent supportive housing (10).

Funding to provide shelter for people experiencing homelessness in the United States (US) comes from federal funding from the US Department of Housing and Urban Development (HUD), which is allocated to local entities through the Continuum of Care program (11). Continuums of Care are highly localized, designed “to promote a community-wide commitment to the goal of ending homelessness,” and consist of stakeholders invested in addressing homelessness, such as non-profit organizations, police, local government representatives, and others. The result of these localized systems is that homelessness resources and policies vary considerably from jurisdiction to jurisdiction.

The number of people experiencing homelessness in any Continuum of Care is determined through a Point in Time (PIT) Count, mandated biannually for all Continuums of Care by HUD to inform HUD’s federal budget allocation (12). A PIT Count is a biannual census of individuals experiencing homelessness on a single night in January (13), and is a required component of all Continuums of Care in their submission of a community-wide, consolidated application for homeless services funding (14). However, the PIT Count data are thought to underrepresent the true total number of individuals experiencing homelessness due to the difficulty of identifying all persons experiencing homelessness within a 24-h time frame (15). The PIT Count is also ill-suited to address question of spatial distribution of homelessness within a city or county, as well as temporal changes in homelessness patterns occurring between biannual counts.

A 2019 report summarizing homelessness policies from 187 cities found that 57% of cities surveyed prohibited camping in at least some public places, 27% prohibited sleeping in at least some public places (such as parks), and 53% prohibited sitting or lying down in at least some public places (16). In San Francisco, California, sleeping is prohibited in parks between the hours of 8:00 PM and 8:00 AM (17), on Port property between the hours of 10:00 PM and 6:00 AM (18), and in vehicles within the City and County of San Francisco between the hours of 10:00 PM and 6:00 AM (19).

In California, homeless service providers are estimated to have only enough beds to house 21% of individuals without shelter (10). In 2019, California was found to have a rate of unsheltered homelessness of 27.4 per 10,000 – the highest in the nation and more than 3.5 times the national average of 6.3 (20). The most recent PIT count conducted in San Francisco was in January 2023, when 4,397 people were counted as unsheltered and 3,185 additional individuals were experiencing homelessness but were sheltered in either transitional housing or an emergency shelter (21). However, San Francisco also reported an overall total of only 3,350 emergency shelter beds at that same time (22), suggesting a deficit of adequate emergency shelter beds for all individuals in need of shelter. Due in part to the lack of emergency beds available for all people experiencing homelessness in San Francisco, the San Francisco-based non-profit organization Coalition on Homelessness filed a lawsuit on September 27, 2022 on the grounds that enforcing local homelessness policies in San Francisco violated the 2018 US Ninth Circuit Court decision Martin v. Boise, which held that the enforcement of anti-camping ordinances violated the eighth Amendment if local jurisdictions failed to provide appropriate places for offenders to sleep (23, 24).

On December 23, 2022, in response to the impending suit, US Magistrate Judge Donna Ryu issued an emergency order granting a preliminary injunction restricting (1) encampment seizing and enclosures (also termed “encampment sweeps” and “homeless encampment resolutions”), and (2) multiple “sit-lie” ordinances and statutes—which outlawed individuals from sitting or lying on public sidewalks—by the San Francisco Police Department and the San Francisco Department of Public Works (25). Judge Ryu’s opinion stated that “for years San Francisco has had a shortfall of shelter beds that numbers in the thousands, and that homeless San Franciscans have not been able to voluntarily access shelter beds since April 2020” (26).

The effect of this recent temporary injunction on the spatial distribution of people experiencing homelessness is not well-understood. A concern raised by injunction opponents has been that the injunction would exacerbate the problem of encampments blocking public places, either increasing the number of encampments in areas that already had them, or expanding the distribution of encampment locations (27, 28). The biannual nature of the PIT Count, the lack of temporal specificity of PIT Count data, and the lack of publicly available geographically-precise data make this data source poorly-suited to answering the question of how populations experiencing homelessness in San Francisco moved during this time. We aimed to answer this question using publicly available data from San Francisco’s 311 system, which contains timestamped and geocoded reports of encampments made by members of the public.

## 2 Materials and methods

Data on 311 reports from the city of San Francisco are publicly available and were obtained from DataSF on April 21, 2024 for the period of July 1st, 2008 through April 21, 2024 (*n* = 6,890,192) (29). A preliminary injunction period was defined as any reports falling between December 23, 2022—when the injunction order was first issues—and October 18, 2023—when the San Francisco Police Department issued a bulletin clarifying enforcement of laws and ordinances for individual experiencing homelessness sitting, lying, or sleeping on public property. The following inclusion criteria were defined: (1) Request Type of “Encampment”; (2) report date fell within the preliminary injunction period December 23, 2022 to October 18, 2023 or 4 years prior, starting from December 23, 2018. Duplicates were identified and removed using Amato et al.’s methodology, by excluding records containing “dup” in the Status Notes or Responsible Agency (30).

Data were processed using R Project for Statistical Computing (RRID: SCR_001905) version 4.3.2 and RStudio (RRID: SCR_000432) version 2023.09.1 + 494. The data were then categorized based on being before versus during the preliminary injunction period and separately categorized as falling within the exact date ranges of the preliminary injunction period, from December 23, 2022 to October 18, 2023, or in prior yearly intervals with the earliest period being from December 23, 2018 to October 18, 2019. Cases were grouped by month and year to calculate the number of encampment cases per 1,000 as well as the percent change in reports monthly. Political boundaries, such as the neighborhoods in San Francisco, represent administrative delineations that may not reflect true spatial interactions. We instead divided San Francisco into a hexagonal grid, providing a uniform approach to spatial analysis that minimizes edge effects and provides consistent areas for comparison (31).

SaTScan is a free software tool designed to analyze spatial, temporal, and space–time scan statistics to detect and evaluate clusters (32). In this study, we used SaTScan to perform two separate spatial analyses of 311 encampment data in San Francisco, applying a continuous Poisson distribution model. The analyses were used to compare the preliminary injunction period with the same period 1 year prior. A circular scanning window was utilized, which systematically moved across San Francisco to test for clusters of varying sizes. For each location and window size, the observed number of encampments was compared to the expected distribution based on the overall data. This comparison generated a likelihood ratio, quantifying the likelihood of observing the data under the null hypothesis of a uniform distribution. The output from SaTScan identified statistically significant clusters of encampments. Only clusters with more than 50 reports were retained. These clusters were assessed to determine whether there were notable differences between the preliminary injunction period and the prior year.

Hot Spot Analysis is a spatial statistics tool used to identify statistically significant spatial clusters (areas with more or less than the expected number of reports) using the Getis-Ord Gi* statistic (33). This calculation evaluates the presence of high values within an area, while also evaluating whether neighboring areas’ values are high as well beyond random chance. A False Discovery Rate (FDR) Correction was applied to account for multiple testing and spatial dependence.

## 3 Results

**Figure 1:**
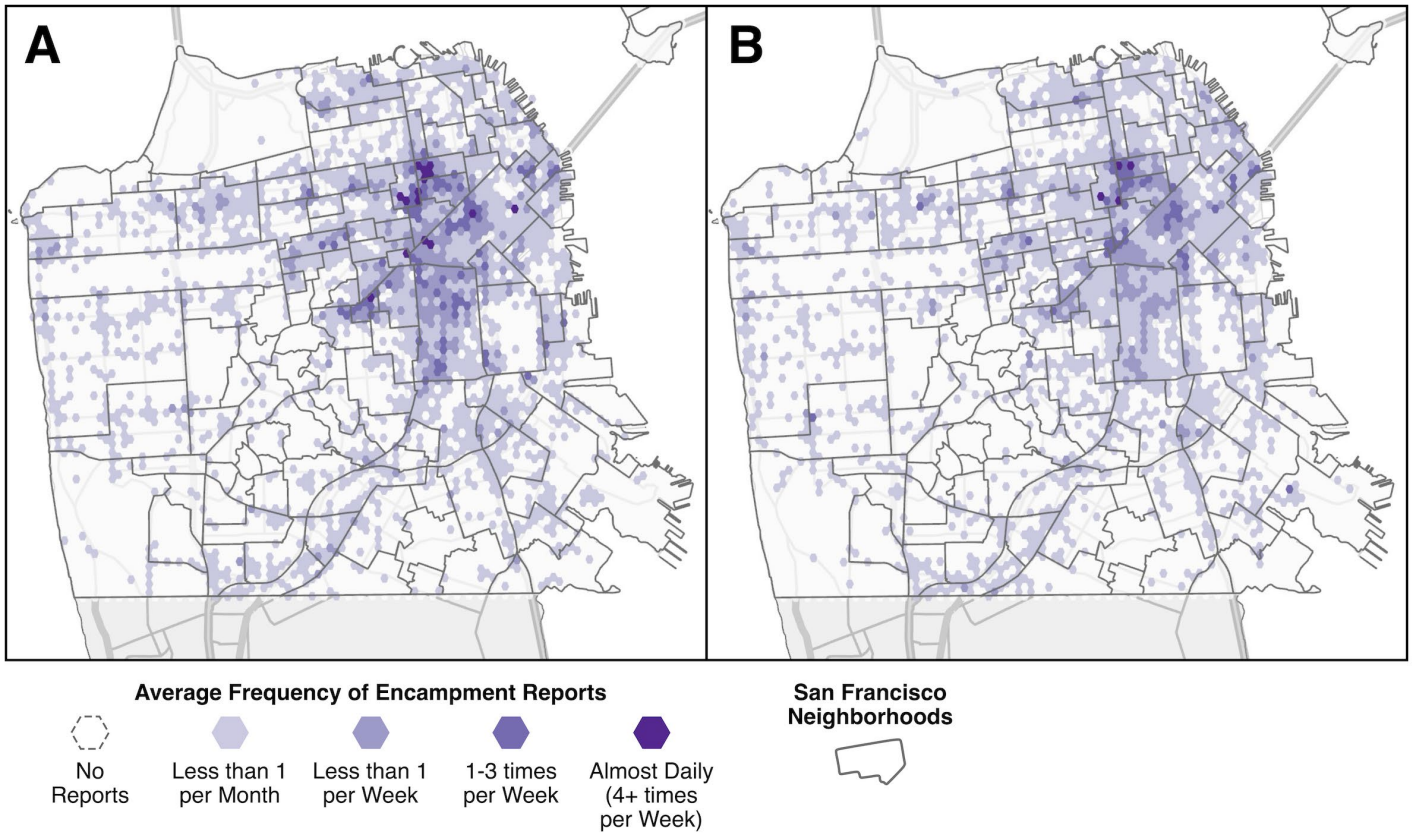
Average frequency of encampment reports to 311 across San Francisco during the study period, with darker shades indicating more frequent reports. Panel **A** shows the pre-injunction period and **B** shows during the preliminary injunction period.

During the preliminary injunction period there were a total of 13,022 encampment reports to 311, compared to 19,079 reports during the same period 1 year prior (see Table 1). This represents a 31.7% decrease in reports during the preliminary injunction period. Reports were made across the city, with the highest average frequency in the northeastern quadrant, both before and during the injunction (see Figure 1). Some areas experienced an average report frequency of four or more reports per week. Most areas saw no change or a decrease in report frequency during the injunction compared to before, with the overall percentage of the city reporting encampments dropping from about 35.5–33.2%.

**Table 1:** Summary of encampment reports to 311 and spatial analyses before vs. during the preliminary injunction period.

	Before injunction	During injunction
Encampment reports	19,079	13,022
Percent of city reporting encampments	35.5%	33.2%
Significant clusters with >50 cases (SaTScan)	3	5
Area covered by clusters	225.5 square kilometers	124.5 square kilometers
Significant hot spots (Getis-Ord)	2	5
Area covered by hot spots	9.25 square kilometers (~9% of city)	9.05 square kilometers (~8.8% of city)

**Figure 2:**
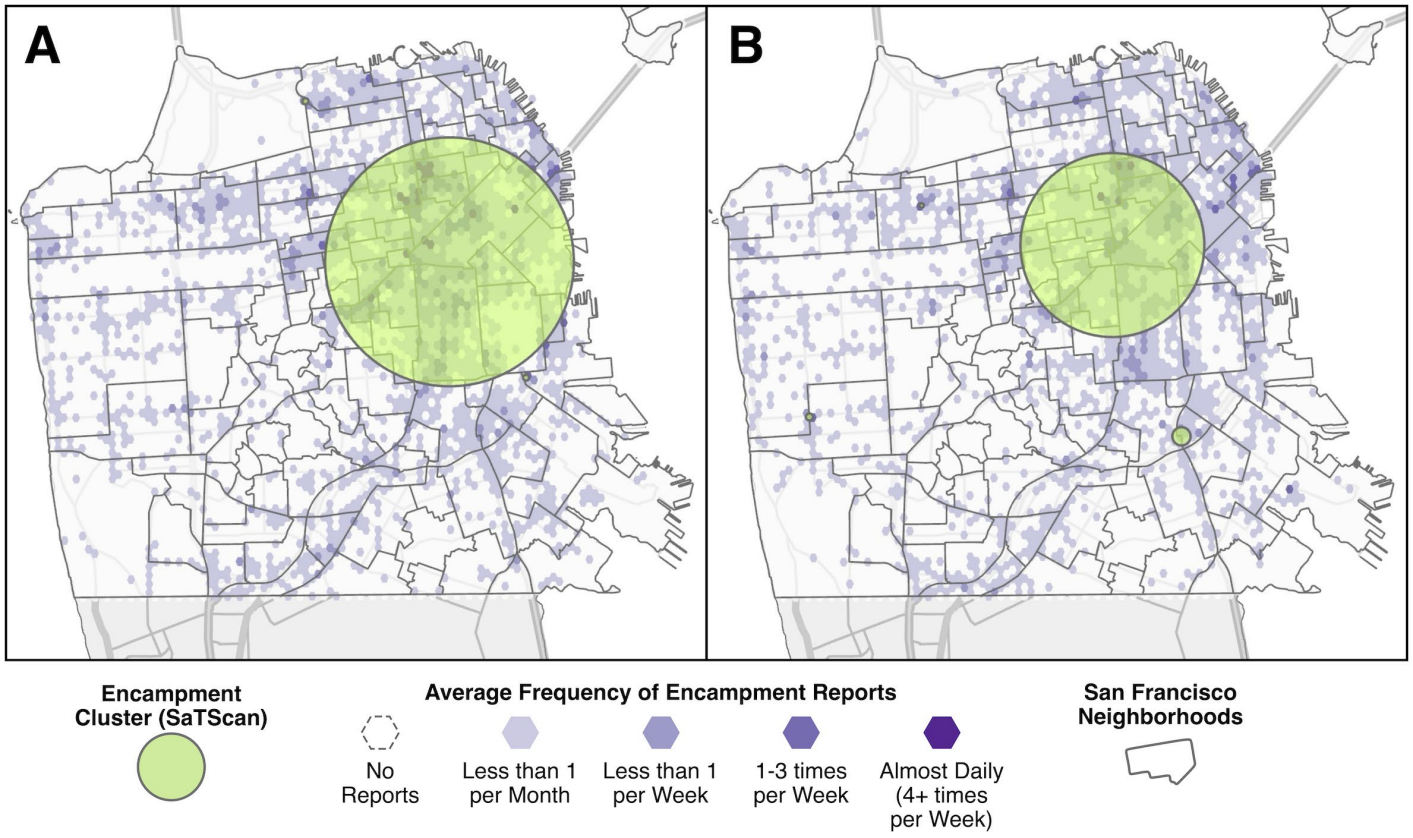
The location and extent of the encampment clusters identified with SaTScan during the pre-injunction period **(A)**, and during the preliminary injunction period **(B)**, with the average frequency of encampment reports shown for comparison.

Cluster analysis with SaTScan produced 3 statistically significant clusters of encampment reports covering approximately 225.5 square kilometers before the injunction. The primary cluster was centered on the border between the South of Market and Mission neighborhoods (see Figure 2). Cluster analysis during the injunction found 5 clusters, but with a reduced overall size covering only 124.5 square kilometers, a 44.8% decrease. The primary cluster was smaller and also somewhat shifted to the northwest, with its center in the Lower Haight neighborhood.

**Figure 3:**
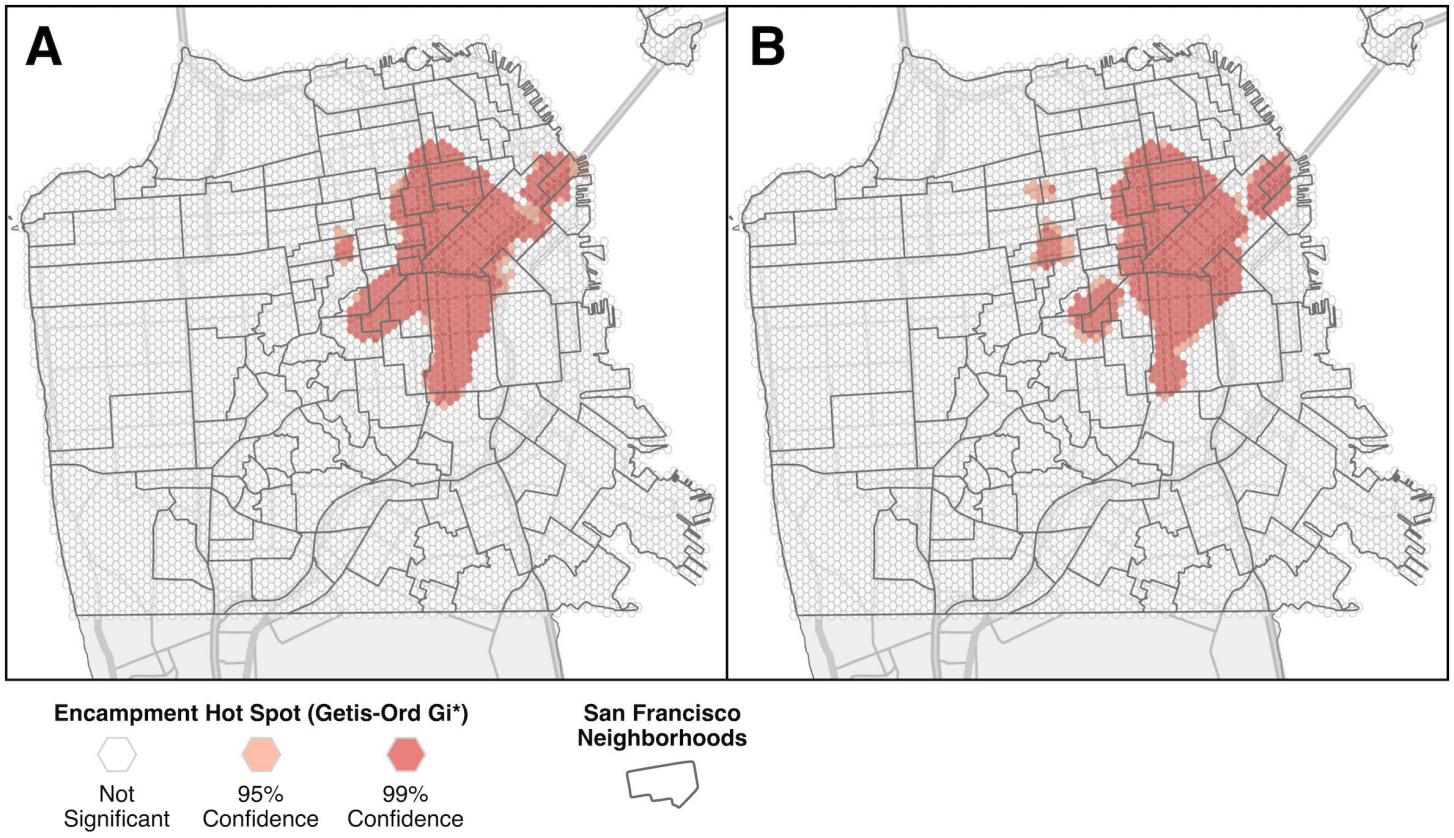
Encampment hot spots identified with the Getis-Ord Gi* test during the pre-injunction period **(A)**, and during the preliminary injunction period **(B)**, shaded according to statistical confidence.

Hot Spot analysis with the Getis-Ord Gi* test for the pre-injunction period found only two contiguous hot spots covering approximately 9.25 square kilometers, almost entirely in the northeast quadrant (see Figure 3). The primary hot spot corresponded to the primary SaTScan cluster, centered roughly in the Civic Center, South of Market, and Mission neighborhoods. The smaller secondary hotspot was centered in the Panhandle neighborhood. Hot Spot analysis during the injunction found 5 separate hot spots, covering 9.05 square kilometers. The primary hot spot was similar to the primary hot spot from the pre-injunction period, only smaller, with new hot spots centered on Rincon Hill and Castro neighborhoods that were previously connected to the primary hotspot. There was also one additional hot spot to the northwest on the border of Laurel Heights/Jordan Park and Lower Pacific Heights.

## 4 Discussion

Homelessness continues to be a serious challenge in many US cities, and policy responses to this challenge remain a contentious issue. While some have investigated the impact of specific policies on the overall homeless population within a city, such as rent control policies in New York City (34), and others have used PIT Count or 311 data to explore the distribution and clustering of homelessness in New York City (35) or Los Angeles (36, 37), this is the first study to combine these approaches to explore the impact of a specific policy on the spatial distribution of homelessness within a city.

One of the concerns raised by opponents of the injunction in San Francisco was that encampments would spread to more parts of the city. We found some evidence both in favor of and refuting this prediction. There was evidence of spread, most obvious in the hot spot analysis (Figure 3), with some areas that had not experienced a significant clustering of encampment reports before the injunction showing significant clustering during the injunction. On the other hand, the same analysis shows an overall reduction in the total proportion of the city with significant clustering, and the absolute number of reports went down by over 30% during the preliminary injunction period as well. This reduction is likely to be at least partially an artifact of underreporting, as public knowledge of the injunction likely led to a decrease in reporting during that time. This means the results during the injunction should be interpreted as likely underestimates of encampment distribution/concentration. Following the Supreme Court decision in the Grants Pass v. Johnson case on June 28, 2024, the US Ninth Circuit Court of Appeals overturned the San Francisco injunction on July 8, 2024 (38, 39). City officials have indicated this decision provides increased flexibility which will likely change enforcement policies, but it is not yet clear to what extent actual enforcement efforts will change (40).

A limitation to this analysis is the fact that sweeps may still have been happening, despite the injunction (28). Whether intentionally disregarding the injunction, exploiting disputed edge case conditions, or accidentally failing to adhere due to misunderstanding or improper training, some encampment sweeps appear to have taken place during the preliminary injunction period. It is not clear how frequent or widespread these were compared to the pre-injunction period, therefore the impact on the results is unknown. Another challenge to interpreting the impact of the injunction is the granularity of the 311 report data. We only know if an encampment is reported in a location, not how long that particular encampment has been there, how large it is, or if the people within it are changing. For example, an area with one or more encampments experiencing sweeps during the pre-injunction period may have had a large number of movements within a small geographic area (e.g., moving around the same block over and over). If this same area were static during the preliminary injunction period, 311 reports between the two time periods would likely indicate no difference during the injunction, despite a substantial difference for the people living in the encampment, as well as those living in housing nearby. More qualitative work is necessary to explore the personal impact of the injunction on people experiencing homelessness in San Francisco during this time, as well as the impact on homes and businesses in the areas where encampments clustered. It is also important to acknowledge that a 311 report of a homeless encampment does not constitute a confirmed encampment, however work in New York City and Los Angeles found strong evidence of concordance between 311 reports of homelessness and official counts (35, 37).

Despite these limitations, our analysis shows a notable shift in 311 encampment reports during the preliminary injunction period compared to the pre-injunction period. The use of crowd-sourced 311 data is an incomplete, but promising, solution to the well-recognized challenge of inadequate and out-of-date data collection on homelessness (35, 41, 42). Most major cities in the US have a 311 service or similar non-emergency citizen reporting system. When cities like San Francisco make these data publicly available, it allows researchers, city managers, and policymakers a near-real-time crowdsourced resource on public sentiment and lived experiences – at least within the constraints imposed by the reporting system.

## 5 Conclusion

Judge Ryu’s preliminary injunction against homeless encampment sweeps in San Francisco remains a controversial policy. Analyses of 311 reports during the preliminary injunction period, when compared to the same time period 1 year prior, suggest the impact of the injunction on the spatial distribution of homeless encampments has been modest but measurable. Encampment reports decreased during the preliminary injunction period and covered less total area in the city, but did increase in intensity in some areas where they had not clustered previously, suggesting both proponents and opponents of the policy were at least partially correct in their predictions. Future work is needed to explore the more personal impact of the injunction on people experiencing homelessness, as well as the economic and other downstream impacts in the most affected areas.

## Data Availability

Publicly available datasets were analyzed in this study. This data can be found at: https://data.sfgov.org/City-Infrastructure/311-Cases/vw6y-z8j6/about_data.
